# Supplementary material for: Chrysanthemum Leaf Ethanol Extract Prevents Obesity and Metabolic Disease in Diet-Induced Obese Mice via Lipid Mobilization in White Adipose Tissue
Source: Nutrients. 2019 Jun 15;11(6):1347. doi: 10.3390/nu11061347 (PMC6628043; doi:10.3390/nu11061347)
Supplement: Supplementary file 1 [file nutrients-11-01347-s001.pdf]

Supplementary Materials

Table S1. Identified flavonoids of chrysanthemum leaf ethanol extract.

No.	Flavonoids name	Content (%)
1	Luteolin	0.008
2	Galuteolin	0.004
3	Luteolin-7-O-glucuronide	0.031
4	Luteolin-7-O-6″-acetyl glucoside	0.002
5	Diosmetin-7-O-rutinoside	0.0004
6	Diosmetin-7-O-glucoside	0.005
7	Acacetin-7-O-rutinoside	0.120
8	Apigenin	0.0006
9	Chlorogenic acid	0.019
10	p-coumaric acid	0.0005

Table S2. Diet composition for animal experiment

Ingredient (g)	ND	HFD	CLE	LU
Casein	200	200	200	200
D,L-methionine	3	3	3	3
Cornstarch	150	111	111	111
Sucrose	500	370	355	370
Cellulose	50	50	50	50
Corn Oil	50	30	30	30
Lard	-	170	170	170
Mineral Mix[1]	35	42	42	42
Vitamin Mix[2]	10	12	12	12
Choline bitartrate	2	2	2	2
Cholesterol	-	10	10	10
TBHQ, antioxidant[3]	0.01	0.04	0.04	0.04
Bioactive compounds			15	0.03
Total (g)	1,000	1,000	1,000	1,000

ND, normal diet (AIN-76); HFD, high-fat diet (45% kcal from fat); CLE, HFD+chrysanthemum leaf (1.5%, w/w); LU, HFD+luteolin (0.003% w/w). [1] AIN-76 Mineral Mixture (g/kg): calcium phosphate, 500; sodium chloride, 74; potassium citrate, 2220; potassium sulfate, 52; magnesium oxide, 24; manganous carbonate, 3.5; ferric citrate, 6; zinc carbonate, 1.6; cupric carbonate, 0.3; potassium iodate, 0.01; sodium celenite, 0.01; chromium potassium sulfate, 0.55; sucrose 118.03 [2]AIN-76 Vitamin Mixture (g/kg): thiamin HCI, 0.6; riboflavin, 0.6; pyridoxine HCI, 0.7; nicotinic acid, 0.003; D-calcium pantothenate, 0.0016; folate, 0.2; D-biotin, 0.02; cyanocobalamin (vitamin B12), 0.001; retinyl palmitate premix, 0.8; DL-alpha tocopherly acetate, premix, 20; cholecalciferol (vitamin D3), 0.0025; menaquinone (vitamin K), 0.05; antioxidant, 0.01; sucrose, finely powdered, 972.8.

Table S3. Classification of functional annotation clusters among up-regulated genes from chrysanthemum leaf ethanol extract and luteolin-responsive genes in epididymal white adipose tissue of C57BL/6J mice fed a high-fat diet.

Group	Term	genes	Count	ES	*p*-value
CLE vs HFD	Glycoprotein	Nt5e, Art3, Abca5, Abcb1a, Abcc9, Abcg2, Clec14a, Clec2d, Cd164l2, Cd19, Cd1d1, Cd209b, Cd300lg, Cd38, Cd46, Dcst1, Eogt, Ephb4, F11r, Flt1, Flt4, Fcamr, Gpr156, Gpr160, Gpr17, Gpr182, Gpr, Mamdc4, Rbmx, Smoc1, St6galnac2, St6galnac5, B3galt2, Galnt15, Galnt18, Acr, Acvr1b, Apcdd1, Adora2a, Adcy4, Adcy5, Amigo2, Adipoq, Adrb1, Adrb2, Adrb3, Acer2, Agtr1a, Agt, Apoe, Bcam, Btc, Bmp4, Bmp6, Bdkrb2, Btnl9, Bche, Cdh13, Calca, Cacna2d1, keratan sulfate Gal-6, Chst1, Car4, Ces1d, Catsperg1, Cp, Cmklr1, Chrnb1, Chrdl1, F2rl1, Cfd, Cnnm3, Dll1, Disp2, Enpp2, Enpp4, Eng, Emcn, Ednrb, Etl4, Efna1, Efnb1, Egf, Emp1, Erbb2, Eva1c, AU021092, Fam20a, Fgf10, Fgfr3, Flrt3), Fbln5, Ffar4, Fzd6, Gabbr1, Gabrr2, Gipr, Gfra4, Gcgr, Gcnt2, Grik5, Gde1, Gpm6a, Glycam1, Grem2, Hfe2, Hs3st3b1, Hrh2, Hsd11b1, Hcn4, Ier3, Insr, Itgb4, Itih4, Icam2, Il15ra, Il2ra, Jag2, Jam2, Jup, Kirrel3, Kdr, Klb, Lamc3, Lama3, Lrrn4, Lgi1, Lect1, Lipg, Lrp5, Ly6c1, Lpar6, Mst1r, Mmp15, Mmp9, Mc5r, Mogat1, Mmrn2, Npr2, Nrg4, Negr1, Nog, Nup210, Olfm2, Pnpla2, Ptch2, Pla2g2d, Plvap, Pdgfd, Podxl, Podn, Pkd2l2, Kcnt1, Kcnc3, Kcne1l, Kcne4, Pcsk5, Ptger3, Ptgfr, Prss55, Ptprb, Ptprm, Ptprr, Ptpru, Robo4, Sgce, Sctr, Sema3f, Sema3g, Sema4c, Sema6a, Serpina1a, Serpina1c, Serpine1, Sdk2, Sigirr, Slit3, Slc1a3, Slc12a2, Slc12a5, Slc19a3, Slc2a3, Slc22a3, Slc4a4, Slc4a11, Slc44a3, Slc52a3, Spock2, S1pr4, Stc2, Sucnr1, Susd4, Synpo, Tex2, Tspan2, Tspan7, Tspan9, Thbs1, Thbs2, Thsd1, Tslp, Akt2, Tfrc, Tgfbr3, Tm4sf1, Tmc7, Tmprss5, Tmem120a, Tmem17, Tmem204, Tmem8b, Ttr, Tinagl1, Tnfsf10, Tie1, Unc5b, Upk1b, Upk3b, Vnn1, Vnn3, Vegfa, Wnt11	224	3.67	1.30E-10
	Insulin signaling pathway	Mknk2, Acaca, Acacb, Eif4ebp1, Irs1, Irs2, Insr, Pygl, Pygm, Pde3b, Pck1, Prkag3, Ppp1r3c, Rps6, Akt2	15	2.28	3.40E-03
	Diet induced thermogenesis	Adrb1, Adrb2, Adrb3, Clic5, Trpv4	5	1.71	1.70E-03
	Positive regulation of glucose import	Adipor2, Adipoq, Irs1, Irs2, Insr, Akt2	6	1.53	1.20E-02
	Positive regulation of fatty acid beta-oxidation	Fabp1, Irs1, Irs2, Akt2	4	1.53	5.40E-03
LU vs HFD	Glycoprotein	Nt5e, Art3, Abca5, Abcb1a, Abcc9, Clec14a, Clec2d, Cd19, Cd209a, Cd209b, Cd209d, Cd300lg, Cd38, Cd46, Emid1, Ephb4, F11r, Gpr156, Gpr160, Gpr182, Gpr4, Gprc5a, Gpihbp1, Kiss1r, Mamdc4, Smoc1, St6galnac2, B3galt2, Galnt18, Vsig2, Apcdd1, Adora2a, Adcy4, Amigo2, Adrb1, Adrb2, Adrb3, Acer2, Agtr1a, Agt, Bcam, B4galnt2, Btc, Bmp4, Bmp6, Bdkrb2, Btnl9, Bche, Cdh13, Cdh5, Cdhr5, Cacna2d3, Car4, Ces1d, Catsperg1, Ccl28, Cxcl9, Chrdl1, Cfd, Crisp1, Disp2, Enpp4, Eng, Emcn, Ednrb, Etl4, Efna1, Efnb1, Epcam, Eva1c, Fam20a, Fgf10, Fgfr3, Fbln5, Ffar4, Fzd6, Glb1l, Gabbr1, Gabrr2, Gipr, Gfra1, Gcgr, Gcnt2, Grik5, Gde1, Gpm6a, Grem2, Hs3st3b1, Hs3st6, Hrh2, Hsd11b1, Hcn4, Ier3, Igfbp2, Itgb4, Icam2, Il15ra, Il2ra, Jag2, Jup, Krt18, Krt8, Kirrel3, Kd, Lamc3, Lama3, Lrrn4, Lrig3, Lipg, Lrp2, Ly6a, Ly6c1, Ltb, Lpar6, Mst1r, Mmp15, Mmp9, Mogat1, Mmrn2, Npr2, Nrg4, Negr1, Nog, Nup210, Olfm2, Ptch2, Pgf, Plvap, Podxl, Pkhd1l1, Pkd2l2, Kcnt1, Kcne1l, Kcne4, Prom1, Ptger3, Prss55, Ptprb, Ptprr, Ptpru, Robo4, Sctr, Sema3f, Sema3g, Sema6a, Serpina1c, Serpina3b, Serpine1, Spint2, Sdk2, Slc1a3, Slc12a2, Slc19a3, Slc2a3, Slc22a3, Slc4a4, Slc4a11, Slc44a3, Slc52a3, Spock2, S1pr1, S1pr4, Susd4, Synpo, Tspan2, Tspan7,	181	5.05	7.50E-14

	Thbs1, Tm4sf1, Tmprss5, Tmem204, Tinagl1, Tnfsf10, Tie1, Upk1b, Upk3b, Vnn1, Vegfa, Wnt10b, Wnt11, Wnt2b, Wnt9a)			
Glutathione metabolic process	Gsta3, Gsta4, Gstm5, Gstt1, Gstt2, Gpx3, Mgst1	7	2.07	3.10E-03
Diet induced thermogenesis	Adrb1, Adrb2, Adrb3, Clic5	4	1.84	5.90E-03
Cellular response to BMP stimulus	Gata6, Bmp4, Bmp6, Heyl, Nog, Spint2	6	1.71	3.50E-03

Differentially expressed genes based on CLE and LU vs HFD comparison according to q-value < 0.05, fold change > 1.5. Functional gene ontology terms enriched among chrysanthemum leaf ethanol extract-responsive genes are clustered according to biological processes (enrichment score>1.5) using DAVID. Term: A detailed item in an annotation source.

Table S4. Primer sequences for genes used for real-time PCR.

Primer	Sequence
Ppara	5′-CCT GAA CAT CGA GTG TCG AAT AT -3′ (forward)<br>5′-GGT GGG CCA GAA TGG CAT CT-3′ (reverse)
Pparg	5′-GAG TGT GAC GAC AAG ATT TG-3′ (forward)<br>5′-GGT GGG CCA GAA TGG CAT CT-3′ (reverse)
Ppargc1a	5′-AAG TGT GGA ACT CTC TGG AAC TG-3′ (forward)<br>5′-GGG TTA TCT TGG TTG GCT TTA TG-3′ (reverse)
Ppargc1b	5′-GGT TCC TGG CTG ACA TTC AC-3′ (forward)<br>5′-GGC ACA TCG AGG GCA GAG-3′ (reverse)
Srebf1a	5′-TAG TCC GAA GCC GGG TGG GCG CCG GCG CCA T-3′ (forward)<br>5′-GAT GTC GTT CAA AAC CGC TGT GTG TCC AGT TC-3′ (reverse)
Srebf1c	5′- GGA GCC ATG GAT TGC ACA TT-3′ (forward)<br>5′-CCT GTC TCA CCC CCA GCA TA-3′ (reverse)
Srebf2	5′-CAC AAT ATC ATT GAA AAG CGC TAC CGG TCC-3′ (forward)<br>5′-TTT TTC TGA TTG GCC AGC TTC AGC ACC ATG-3′ (reverse)
Cpt1a	5′-ATC TGG ATG GCT ATG GTC AAG GTC-3′ (forward)<br>5′-GTG CTG TCA TGC GTT GGA AGT C-3′ (reverse)
Cpt2	5′-GCC TGC TGT TGC GTG ACT G-3′ (forward)<br>5′-TGG TGG GTA CGA TGC TGT GC-3′ (reverse)
Scd1	5′-CCC CTG CGG ATC TTC CTT AT-3′ (forward)<br>5′-AGG GTC GGC GTG TGT TTC T-3′ (reverse)
Fasn	5′-GCT GCG GAA ACT TCA GGA AAT-3′ (forward)<br>5′-AGAGAC GTG TCA CTC CTG GAC TT-3′ (reverse)
Acaca1	5′-GCC TCT TCC TGA CAA ACG AG-3′ (forward)<br>5′-TGA CTG CCG AAA CAT CTC TG-3′ (reverse)
Sirt1	5′-TGT GAA GTT ACT GCA GGA GTG TAA A-3′ (forward)<br>5′-GCA TAG ATA CCG TCT CTT GAT CTG AA-3′ (reverse)
Ucp1	5′-AGA TCT TCT CAG CCG GAG TTT-3′ (forward)<br>5′-CTG TAC AGT TTC GGC AAT CCT-3′ (reverse)
Cox8b	5′-TGT GGG GAT CTC AGC CAT AGT-3′ (forward)<br>5′-AGT GGG CTA AGA CCC ATC CTG-3′ (reverse)
Cidea	5′-TTT CAA ACC ATG ACC GAA GTA GCC-3′ (forward)<br>5′-CCT CCA GCA CCA GCG TAA CC-3′ (reverse)
Adrb3	5′- ACC AAC GTG TTC GTG ACT-3′ (forward)<br>5′- ACA GCT AGG TAG CGG TCC-3′ (reverse)
Fgf21	5′- ATG GAA TGG ATG AGA TCT AGA GTT GG-3′ (forward)<br>5′- TCT TGG TGG TCA TCT GTG TAG AGG-3′ (reverse)
Acox1	5′- CCC AAC TGT GAC TTC CAT T-3′ (forward)<br>5′- GGC ATG TAA CCC GTA GCA CT-3′(reverse)
Acadm	5′-CAA GTT CAA CGG CAC AGT CAA GG-3′ (forward)<br>5′-ACA TAC TCA GCA CCA GCA TCA CC-3′ (reverse)
Acadl	5′-TCA CCA ACC GTG AAG CTC GA-3′ (forward)<br>5′-CCA AAA AGA GGC TAA TGC CAT G-3′ (reverse)
Adipoq	5′-GCA CTG GCA AGT TCT ACT GCA A (forward)<br>5′-GTA GGT GAA GAG AAC GGC CTT GT (reverse)
Tnfa	5′-AAA GAC ACC ATG AGC ACA GAA AGC-3′ (forward)<br>5′-GCC ACA AGC AGG AAT GAG AAG AG-3′ (reverse)
Tlr2	5′-GAG CAT CCG AAT TGC ATC AC-3′ (forward)

	5′-TAT GGC CAC CAA GAT CCA GA-3′ (reverse)
Tlr4	5′-AAG AGC CGG AAG GTT ATT GTG-3′ (forward)<br>5′-CCC ATT CCA GGT AGG TGT TTC-3′ (reverse)
36B4	5′-CAG ATT GGC TAC CCA ACT GTT-3′ (forward)<br>5′-GGG AAG GTG TAA TCC GTC TCC-3′ (reverse)
